# Comparative Effectiveness of CURB-65 and qSOFA Scores in Predicting Pneumonia Outcomes: A Systematic Review

**DOI:** 10.7759/cureus.71394

**Published:** 2024-10-13

**Authors:** Abdulhadi Gelaidan, Mohanad Almaimani, Yara A Alorfi, Anas Alqahtani, Nawaf G Alaklabi, Shahad M Alshamrani, Raneem Rambo, Joury A Mujahed, Ruba Y Alsulami, Mohammed Namenkani

**Affiliations:** 1 Internal Medicine, University of Jeddah, Jeddah, SAU; 2 Medicine and Surgery, University of Jeddah, Jeddah, SAU; 3 Medicine, University of Jeddah, Jeddah, SAU

**Keywords:** evidence-based recommendations, icu admission, pneumonia severity assessment, prediction, sensitivity and specificity, sepsis, short-term mortality

## Abstract

Pneumonia is a leading cause of hospitalization and mortality worldwide, often progressing to sepsis, making early accurate severity assessment crucial for effective clinical decision-making. This systematic review compares the CURB-65 (confusion, urea, respiratory rate, blood pressure, and age ≥65 years) and qSOFA scoring systems in predicting pneumonia outcomes, including short-term mortality and ICU admission, to provide evidence-based recommendations for their clinical application. A comprehensive search was conducted across multiple databases, including PubMed, Medline, Web of Science, Google Scholar, Cochrane Library, and BMJ Journals, using specific keywords related to pneumonia and the scoring systems. Eligible studies included adult patients diagnosed with community-acquired, hospital-acquired, or healthcare-associated pneumonia (HAP), where CURB-65 or qSOFA scores were calculated within 24 hours of admission. Data extraction focused on study characteristics, patient demographics, and outcome measures, with quantitative synthesis comparing the predictive performance of the two scores. Sensitivity, specificity, and area under the ROC curve (AUC) values were assessed, and potential sources of heterogeneity and publication bias were examined. The analysis included 22 studies with a total of 25,846 participants, revealing varying predictive accuracy across different settings. CURB-65 demonstrated superior sensitivity (76.52%) and AUC (0.747) for mortality prediction, making it a more reliable tool for identifying high-risk pneumonia patients who require intensive management. Conversely, qSOFA exhibited superior specificity (86.08%) and better performance in predicting ICU admissions, with an AUC of 0.714, highlighting its utility in identifying patients who may need critical care interventions. This systematic review underscores the strengths and limitations of both CURB-65 and qSOFA in predicting pneumonia outcomes. While CURB-65 is more effective for mortality prediction, qSOFA excels in predicting ICU admissions. A combined approach leveraging both scoring systems could enhance patient assessment and management. Further research with larger, prospective studies is recommended to validate these findings and optimize the clinical use of CURB-65 and qSOFA in pneumonia management.

## Introduction and background

Pneumonia is a major cause of hospitalization and mortality across the world, often complicated by sepsis [1]. The definitions and distinctions among community-acquired pneumonia (CAP), healthcare-associated pneumonia (HCAP), and hospital-acquired pneumonia (HAP) are well articulated in the literature, particularly in a study published by the National Center for Biotechnology Information (NCBI). CAP is characterized as pneumonia occurring in individuals without recent hospitalization or significant healthcare exposure, while HCAP refers to pneumonia developing in patients with specific healthcare risk factors prior to hospitalization. HAP is defined as pneumonia that occurs more than 48 hours after hospital admission and is often associated with more resistant pathogens. This categorization is crucial for guiding appropriate antimicrobial therapy and improving patient outcomes [2]. Key risk factors include age, with children under five and adults over 65 being at the highest risk. Chronic health conditions such as heart disease, diabetes, and chronic lung diseases also elevate the likelihood of developing pneumonia. Additionally, lifestyle involving smoking and excessive alcohol consumption can impair immune function, increasing susceptibility to infections. Individuals with weakened immune systems, whether due to medical conditions or treatments like chemotherapy, face a greater risk of pneumonia and its complications [3]. According to the World Health Organization (WHO), pneumonia was responsible for nearly 15% of all deaths among children under five years old in 2019, which translates to approximately 740180 children [4]. Pneumonia is also a substantial concern among the older population. The incidence of CAP is around three episodes per 1000 person-years in adults between 65 and 69; however, this number rises significantly to almost 22 episodes per 1000 person-years in individuals aged between 85 and 89 [5].

The severity of pneumonia varies widely, ranging from mild, community-acquired infections to severe cases that necessitate intensive care. The clinical outcomes of pneumonia are influenced by several factors, such as age, comorbidities, the causative pathogen, and the appropriateness of the medical approach [6]. To help manage pneumonia, several scoring systems have been introduced to predict pneumonia outcomes. These scoring systems integrate various clinical parameters to estimate the severity of the disease and adverse outcomes. Although these tools are widely used in clinical practice, their efficacy is often challenged. For example, Noguchi et al., in their systematic review and meta-analysis, reported that scoring tools are not effective in predicting mortality in HCAP [7]. The CURB-65 (confusion, urea, respiratory rate, blood pressure, and age ≥65 years) and qSOFA scoring systems are essential for evaluating the severity of CAP and guiding clinical decisions regarding hospital admission and critical care needs. The CURB-65 score includes five parameters: confusion (1 point), urea, >7 mmol/L (1 point); respiratory rate, ≥30 breaths per minute (1 point); blood pressure (systolic <90 mmHg or diastolic ≤60 mmHg, 1 point); and Age ≥65 years (1 point). Scores are interpreted as follows: 0-1 indicates low risk (suitable for outpatient treatment), 2 indicates moderate risk (consideration for inpatient treatment), and scores of 3 or higher indicate high risk, warranting hospital admission, potential ICU transfer, and closer monitoring due to a mortality risk that can exceed 40% for scores of 4-5 [8]. The qSOFA score is a clinical tool used to identify patients at risk of poor outcomes due to infection, particularly in emergency settings. It consists of three parameters: respiratory rate (≥22 breaths per minute, 1 point), altered mental status (Glasgow Coma Scale <15, 1 point), and systolic blood pressure (≤100 mmHg, 1 point). Each parameter contributes 1 point, leading to a total score ranging from 0 to 3. A score of 2 or more indicates a higher risk of mortality and poor outcomes, suggesting the need for closer monitoring or possible admission to the intensive care unit (ICU). Mortality rates associated with qSOFA scores show a significant correlation: approximately 11.9% for a score of 0, 17.9% for a score of 1, 30.1% for a score of 2, and up to 47.2% for a score of 3 [9]. The most commonly used scoring tools for pneumonia include the pneumonia severity index (PSI), CURB-65, and the qSOFA scores. The qSOFA score is strongly associated with mortality prediction in pneumonia patients, with a pooled risk ratio of 3.35 for a score ≥2 [10]. It is also effective in predicting ICU admission. The CURB-65 score is widely used for assessing pneumonia severity. However, the superiority of one tool over the other remains controversial [11]. Therefore, the present systematic review was conducted to identify gaps in the existing literature and find the comparative effectiveness of CURB-65 and qSOFA scores in predicting pneumonia outcomes. This research will also provide evidence-based recommendations to clinicians for the future application of these scoring tools. 

Methods

Study Design and Setting

This systematic review focused on adult patients (≥18 years) diagnosed with pneumonia, including CAP, HAP, and HCAP. The primary intervention involved calculating the CURB-65 and qSOFA scores at the time of or within 24 hours of admission for each patient. The aim was to compare the predictive performance of these two scoring systems in determining clinical outcomes for pneumonia patients. The review encompassed studies conducted in various healthcare settings, including primary, secondary, and tertiary hospitals, within the ICU setting, where both CURB-65 and qSOFA scoring systems were utilized to assess pneumonia severity.

Outcomes

The primary outcome of interest was short-term mortality, defined as death within 30 days of admission. Secondary outcomes included the rate of appropriate ICU admission.

Search Strategy

A comprehensive search started in February was conducted across multiple bibliographic databases, including PubMed, Medline, Web of Science, Google Scholar, Cochrane Library, and BMJ Journals. Specific keywords such as "Pneumonia," "CURB-65 scoring system," and "qSOFA scoring system" were used. Forward and backward citation searches were also performed to identify additional relevant studies and only English language studies were included.

Inclusion and Exclusion Criteria

Eligible studies included adult patients aged 18 years or older diagnosed with CAP, HAP, or HCAP, utilizing either CURB-65 or qSOFA scoring systems for severity assessment at the time of or within 24 hours of admission. Studies were included if they compared the predictive performance of these scores regarding outcomes such as short-term mortality, ICU admission, mechanical ventilation requirement, and length of hospital stay. We included randomized controlled trials (RCTs), retrospective studies, prospective studies, case series, and case-control studies. Additionally, review articles and cross-sectional studies were excluded. Exclusions applied to studies involving pediatric populations, COVID-19 pneumonia, aspiration pneumonia, and pneumonitis, as well as those not reporting relevant clinical outcomes or not utilizing the specified scoring systems. 

Data Extraction and Screening

The results from electronic database queries were imported into Rayyan Software to facilitate screening and selection. Three authors independently reviewed the titles and abstracts of all identified studies to determine potential eligibility. Full-text articles of studies meeting the inclusion criteria were obtained and assessed independently by the same authors, with any discrepancies resolved by consulting a senior author. The screening process, including reasons for exclusion, was meticulously documented, utilizing a PRISMA flowchart.

Quality Assessment

The quality of the included studies was appraised using the MINOR (Methodological Index for Non-Randomized Studies) tool, ensuring relevant content and methodological rigor were evaluated. 

Data Synthesis

A systematic approach was employed to extract relevant study characteristics, patient demographics, and outcome measures. Quantitative synthesis, including meta-analysis where appropriate, compared the predictive performance of CURB-65 and qSOFA scores. Heterogeneity among studies was assessed, and subgroup analyses explored potential sources of variation. Publication bias was evaluated, and narrative synthesis complemented the quantitative findings, summarizing trends and variations across studies. Sensitivity analyses assessed the robustness of the results, culminating in a comprehensive summary that considered the strengths, limitations, and implications of the evidence regarding the comparative effectiveness of CURB-65 and qSOFA scores in predicting pneumonia outcomes.

## Review

Results

**Figure 1:**
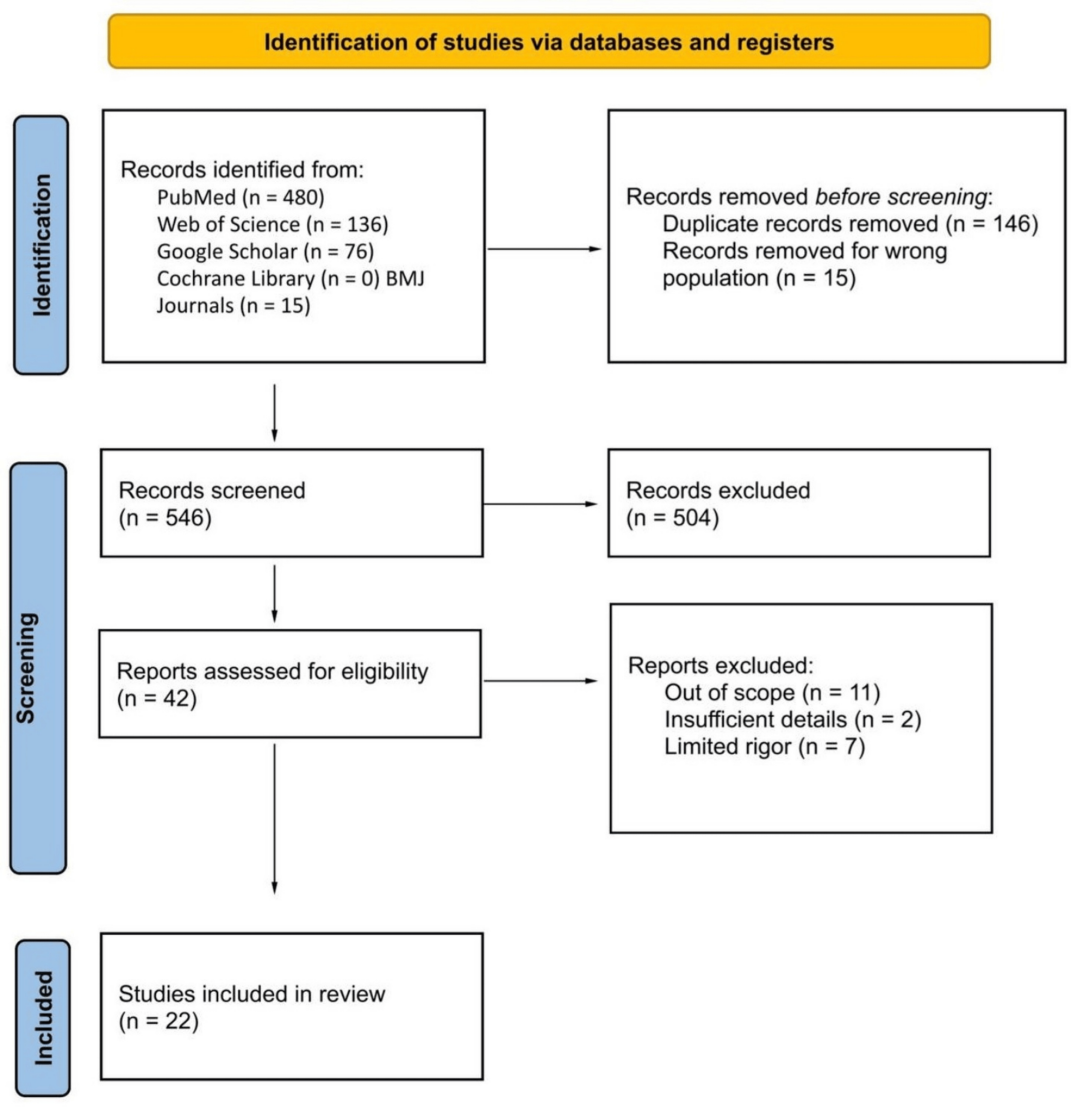
PRISMA flow diagram of the search for key question

The literature search provided 707 number of studies from PubMed (n = 480), Web of Science (n = 136), Google Scholar (n = 76), Cochrane Library (n = 0), and BMJ Journals (n = 15). During the screening, 675 records were removed based on title and abstract. Thirty-two articles were included in the full-length screening. Following a thorough assessment of full-length articles, 22 articles were included in this systematic review, as shown in Figure 1.

A total of 25,846 participants were included in the studies in this systematic review. Most of the studies were retrospective (15/22), followed by prospective ones (6/22), with one being a cohort study. The number of male participants varied from 45.93% to 77.21%, with an average of 59.54% in the included studies. The average age of the participants fell between 60 and 84.5 years. ICU admission rate varied widely from 5% to 100% in some studies, with an average of 22.14%. The survival rate of participants spanned from 73.5% to 95.3%. The average survival rate of all the included studies was 87.21%, as shown in Table 1.

**Table 1:** Demographic details, ICU admission rates, hospital days, and survival rates in patients in included studies ICU, intensive care unit; IQR, intraquartile range; N/A, not applicaple

Study authors	Study design	Participants	Male (%)	Age (years)	ICU admission	Hospital days	Survival rate
Lv, et al., 2021 [11]	Retrospective study	1044	55.36	≥65 years	9.7%	N/A	86.39%
Hincapié, et al., 2021 [12]	Prospective study	Cohorts 1, 2, and 3 included 158, 745, and 207	Cohort 1: 65.8; cohort 2: 51.1; cohort 3: 55.6	Cohort 1,2,3, respectively, the median age was 70 (IQR = 56 - 81), 66 (IQR = 54 - 77) and 60 (IQR = 44-75), respectively.	83 (52.5%), 324 (43.5%), 53 (25.6%) in cohorts 1, 2, and 3 respectively	N/A	Cohort 1: 107 (67.7%) Cohort 2: 617 (82.2%) Cohort 3: 169 (81.6%)
Lv, et al., 2022 [13]	Retrospective study	1025, survivor group: 895, non-survivor group: 130	Survivor group: 55.08; non-survivor group: 58.46	Survivor group: 79.2 ± 8.8; non-survivor group: 84.1 ± 8.1	N/A	N/A	87.31%
Zhang, et al., 2020 [14]	Retrospective study	742	62.26	ICU: 70.40 ± 13.46, non-ICU: 68.60 ± 13.74	23.45%	28	N/A
Tokioka, et al., 2018 [15]	Prospective study	1045	71.4	77 (68 - 83)	7.9%	11	93.9%
Song, et al., 2019 [16]	Retrospective study	443	57.11	65.5 ± 15.1	9.7%	2 - 12	90%
Muller, et al., 2017 [17]	Retrospective study	527	64.51	66	22%	7 (4 - 12)	93.9%
English, et al., 2019 [18]	Prospective study	6013	45.93	67	17.62%	N/A	N/A
Yamazaki, et al., 2021 [19]	Retrospective study	79	77.21	74.6 ± 5.7	N/A	25.8 ± 40.7	84.9%
Carmo, et al., 2021 [20]	Prospective study	200	48	81 (67 - 90)	100%	N/A	N/A
Grudzinska, et al., 2019 [21]	Retrospective study	1545	50.80	76 (63 - 85)	6.4	6.99 (3.65 - 14.1)	N/A
Asaia, et al., 2019 [22]	Retrospective study	406	63	79 (19 - 103)	N/A	N/A	N/A
Yoo, et al., 2020 [23]	Retrospective study	624	65.54	74.0 (61.0 - 81.0)	N/A	8.0 (5.0 - 15.0)	88.8
Beğenen, et al., 2020 [24]	Prospective study	400	55.25	60.39 (18 - 94)	N/A	-	83%
Ranzani, et a., 2017 [25]	Cohort study	6,874	62.2	66.1 ± 19	13.8%	7 (4 - 10)	93.6%
Wen, et al., 2020 [26]	Retrospective study	223	69.5	30-day survivors (73.5) and 30-day non-survivors (79.0)	N/A	N/A	N/A
Zhou, et al., 2020 [27]	Retrospective study	336	63.39	76	18.2%	N/A	73.5%
Spagnolello, et al., 2021 [28]	Retrospective study	505	52.67	71	13.6%	N/A	95.3%
Guo, et al., 2022 [29]	Prospective study	2116	N/A	N/A	N/A	N/A	93.57%
Özyurt, et al., 2022 [30]	Retrospective study	100	60	84.5 (62 - 90) for those who died and 75 (19 - 96) for those who survived	N/A	N/A	88%
Zhou, et al., 2019 [31]	Retrospective study	350	62	78 (65 - 84)	N/A	28	78.9%
Tripon, et al., 2021 [32]	Retrospective study	139	56.8	58.73 ± 18.84	5%	7.35 ± 3.41	N/A

Table 2 presents the sensitivity, specificity, and AUC values from qSOFA and CURB-65. Seventeen studies reported the sensitivity for qSOFA. qSOFA ≥2 criteria were used in 10 studies. The sensitivity of qSOFA to predict mortality ranged from 16.6% to 98.5%, with an average sensitivity of 56.75%. Similarly, the sensitivity for predicting ICU admission ranged from 34.2% to 86.27%. The average sensitivity was 58.81%. Regarding mortality specificity, studies using qSOFA ≥2 reported an average specificity of 86.08% (75%-96.6%). The specificity for ICU admission was 75.5% (54.6%-88.5%). The average sensitivity of CURB-65 for predicting mortality in studies with CURB-65 ≥2 was 76.52%, whereas for CURB ≥3, this value was 72.08%. This outcome was reported by five and six studies, respectively. The sensitivity of CURB-65 for predicting ICU admission was 37.32% for studies with CURB ≥3. Only two studies reported ICU admission prediction outcomes. Regarding specificity, five studies used CURB-65 ≥2 parameters and reported an average mortality specificity of 54.28% in these studies. Six studies reported CURB ≥3 parameters and reported specificity for mortality prediction of 73.2%. Two studies with CURB-65 ≥3 reported specificity for ICU admission prediction. The average specificity was 82.72%.

**Table 2:** Sensitivity, specificity, and AUC values for qSOFA and CURB-65 CURB-65, scoring system based on confusion, urea, respiratory rate, blood pressure, and age ≥65 years; AUC, area under the curve, a statistical measure used to evaluate the performance of diagnostic tests

Authors	qSOFA		CURB-65		AUC for mortality prediction		AUC for ICU admission prediction	
	Sensitivity (%)	Specificity (%)	Sensitivity (%)	Specificity (%)	qSOFA	CURB-65	qSOFA	CURB-65
Lv, et al., 2021 [11]	ICU admission: 86.27; mortality: 80.99	ICU admission: 81.83; mortality: 84.02	ICU admission: 96.08; mortality: 62.68	ICU admission: 56.54; mortality: 92.45	0.844	0.927	0.866	0.922
Hincapié, et al., 2021 [12]	ICU admission: 55.4	Mortality: 43.9	Mortality: 5 8.8	ICU need: 93.5; mortality: 93.4	0.60	0.66	0.59	0.43
Lv, et al., 2022 [13]	N/A	N/A	N/A	N/A	Training cohort: 0.71; validation cohort: 0.62	Training cohort: 0.86; validation cohort: 0.84	N/A	N/A
Zhang, et al., 2020 [14]	qSOFA = 1; mortality: 54.18; ICU admission: 70.11	qSOFA=1; mortality: 64.56; ICU admission: 65.32	CURB-65 = 2; mortality: 58.53; CURB-65 = 3; ICU admission: 44.25	CURB-65 = 2; mortality: 62.30; CURB-65 = 3; ICU admission: 85.74	0.602	0.614	0.712	0.705
Tokioka, et al., 2018 [15]	qSOFA ≥2; mortality: 39.1; ICU admission: 41	qSOFA ≥2; mortality: 87.8; ICU admission: 88.5	CURB-65 ≥2; mortality: 87.5; ICU admission: 83.1	CURB-65 ≥2; mortality: 41; ICU admission: 41.2	0.69	0.75	0.76	0.73
Song, et al., 2019 [16]	qSOFA ≥2; mortality: 29.6	qSOFA ≥2; mortality: 95	CURB-65 ≥3; mortality: 29.6	CURB-65 ≥3; mortality: 95	0.720	0.652	N/A	N/A
Muller, et al., 2017 [17]	qSOFA ≥1; ICU admission: 34.2; mortality: 15.5	qSOFA ≥1; ICU admission: 87.5, mortality: 87.5	CURB-65 ≥1; ICU admission: 28.3; mortality: 15.9	CURB-65 ≥1; ICU admission: 77.8; mortality: 92.1	0.58	0.650	0.66	0.586
English, et al., 2019 [18]	Mortality: 23.8	Mortality: 94.3	Mortality: 18.7	Mortality: 95.2	0.600	0.643	N/A	N/A
Yamazaki, et al., 2021 [19]	qSOFA ≥ 2; mortality: 98.5	qSOFA ≥2; mortality: 75	CURB-65 ≥3; mortality: 98.5	CURB-65 ≥3; mortality: 66.6	0.692	0.712	N/A	N/A
Carmo, et al., 2021 [20]	N/A	N/A	N/A	N/A	0.64	0.65	N/A	N/A
Grudzinska, et al., 2019 [21]	qSOFA ≥ 2; mortality: 40.3	qSOFA ≥2; mortality: 79.9	CURB-65 ≥2; mortality: 85	CURB-65 ≥2; mortality: 40.1	0.62	0.69	N/A	N/A
Asaia, et al., 2019 [22]	qSOFA ≥2; mortality: 56.3	qSOFA ≥2: mortality: 82.2	CURB-65 ≥3; mortality: 47.4	CURB-65 ≥3; mortality: 73.6	N/A	N/A	N/A	N/A
Yoo, et al., 2020 [23]	N/A	N/A	N/A	N/A	N/A	N/A	N/A	N/A
Beğenen, et al., 2020 [24]	qSOFA ≥2; mortality: 60	qSOFA ≥2; mortality: 84.85	CURB-65 >2; mortality: 60	CURB-65 >2; mortality: 87.12	0.769	0.817	N/A	N/A
Ranzani, et al., 2017 [25]	qSOFA ≥2; mortality: 50	qSOFA ≥2; mortality: 81	CURB-65 ≥3; mortality: 78	CURB-65 ≥3; mortality: 60	0.697	0.746	N/A	N/A
Wen, et al., 2020 [26]	N/A	N/A	N/A	N/A	0.767	0.744	N/A	N/A
Zhou, et al., 2020 [27]	qSOFA ≥2; mortality: 91	qSOFA ≥2; mortality: 53.8	CURB-65 ≥3; mortality: 75.3	CURB-65 ≥3; mortality: 61.9	0.807	0.744	0.822	0.774
Spagnolello, et al., 2021 [28]	qSOFA ≥2; mortality: 83.3, qSOFA ≥1; ICU admission: 62.5	qSOFA ≥2; mortality: 90.6, qSOFA ≥1; ICU admission: 54.6	CURB-65 ≥3; mortality: 91.7; ICU admission: 30.4	CURB-65 ≥3; mortality: 82.1; ICU admission: 79.7	0.909	0.923	0.585	0.570
Guo, et al., 2022 [29]	qSOFA ≥2; mortality: 59.6	qSOFA ≥2; mortality: 88.30	N/A	N/A	0.868	0.919	N/A	N/A
Özyurt, et al., 2022 [30]	qSOFA ≥1; mortality: 75; qSOFA ≥2; mortality: 16.6	qSOFA ≥1; mortality: 73.8; qSOFA ≥2; mortality: 96.6	CURB-65 ≥2; mortality: 91.6	CURB-65 ≥2; mortality: 40.9	N/A	N/A	N/A	N/A
Zhou, et al., 2019 [31]	qSOFA ≥1.5; mortality: 89.2	qSOFA ≥1.5; mortality: 71.7	CURB-65 ≥2.5; mortality: 82.4	CURB-65 ≥2.5; mortality: 68.8	0.861	0.808	N/A	N/A
Tripon, et al., 2021 [32]	N/A	N/A	N/A	N/A	0.542	0.586	N/A	N/A

The AUC for mortality prediction was reported by 19 studies. For qSOFA, the AUC for mortality ranged from 0.542 to 0.909, whereas the average value was 0.707. The average AUC for mortality prediction for CURB-65 was 0.747 (0.586-0.927). While comparing qSOFA with CURB-65, it was revealed that only four studies showed the superiority of qSOFA over CURB-65 regarding mortality prediction, whereas 15 studies favored CURB-65. Regarding AUC for ICU admission prediction, only seven studies reported this outcome. Out of these studies, only one study showed the superiority of CURB-65 over qSOFA, whereas six studies showed better AUC for qSOFA. The average value of qSOFA was 0.714 (0.585-0.866), whereas it was 0.674 (0.43-0.922) for CURB-65.

Discussion

Pneumonia is considered a significant contributor to sepsis and is associated with high mortality. The outcomes of pneumonia pose tremendous challenges for clinicians. This hinders their ability to diagnose pneumonia early and provide appropriate care. Early management of pneumonia is considered a key factor in improving patient outcomes. This systematic review compares the effectiveness of the CURB-65 and qSOFA scoring systems in predicting mortality and ICU admission among pneumonia patients. The data from 25,846 patients across 22 studies revealed that CURB-65 is superior to qSOFA in predicting mortality in pneumonia patients; however, qSOFA was superior in predicting ICU admission. Notably, the sensitivity of CURB-65 at score category ≥2 was much higher for mortality compared to qSOFA (76.52% vs. 56.75%). However, qSOFA demonstrated greater specificity when compared to CURB-65 (86.08% vs. 54.28%). To the best of our knowledge, this is the first systematic review to compare the predictive effectiveness of qSOFA and CURB-65 in pneumonia patients. 

The qSOFA criteria include three vital signs: blood pressure, respiratory rate, and mentation. The assessment of altered mentation is more straightforward in qSOFA than in other tools [33]. On the other hand, CURB-65 is straightforward, with just five criteria: confusion, urea levels, respiratory rate, blood pressure, and age over 65 years. Despite its simplicity, this score does not perform well in young adult populations [34]. The findings of the present systematic review align with the systematic review by Marx, et al., who reported that CURB-65 and qSOFA are the best predictors of mortality [33]. In their systematic review, they did not find a significant difference between CURB-65 and qSOFA regarding AUC for mortality. Although CURB-65 has not been compared with qSOFA previously in any systematic review, its comparison with other tools has demonstrated its promising efficacy. For example, Zaki, et al., while comparing PSI and CURB-65 in their meta-analysis, reported that CURB-65 was better at predicting mortality in severe pneumonia cases [35]. Furthermore, the CURB-65 score effectively predicted 30-day mortality, the need for mechanical ventilation, and hospital admission. Additionally, the time to achieve clinical stability was associated with the CURB-65 score [35].

Müller, et al. found that a qSOFA score of 2 or higher was linked to a higher risk of death in patients with pneumonia. They also demonstrated that qSOFA and CURB-65 had comparable abilities to predict in-hospital mortality in pneumonia patients, with qSOFA showing better performance than CURB-65 for predicting ICU admissions [16]. However, the overall comparison between qSOFA and other traditional scoring systems is still debatable. In contrast, Ranzani, et al. reported that CRB and CURB-65 had superior predictive performance compared to qSOFA in patients with CAP [25].

The sensitivity and specificity of qSOFA identified in the present systematic review align with the findings of a systematic review by Jiang, et al., which assessed the role of qSOFA in mortality prediction [10]. Their systematic review included six studies that revealed that a qSOFA score ≥2 was associated with a high risk of mortality in pneumonia patients. The sensitivity and specificity reported in their systematic review were 0.43 and 0.56, respectively. In contrast with our findings, a retrospective study reported that qSOFA has better performance than CURB-65 regarding mortality. However, this study was conducted in COVID-19 patients and had a small number of participants [36].

Strengths and limitations

The main strength of this systematic review is that it is the first systematic review to assess the comparative efficacy of qSOFA and CURB-65. Furthermore, a systematic search in various databases and registries was undertaken to ensure that all articles that met the inclusion criteria were included in this systematic review. There are several limitations of this systematic as well that should be considered while interpreting the findings. The predominance of retrospective studies may introduce biases related to data collection and patient selection. Furthermore, in this systematic review, only descriptive details of the included studies are provided, and no meta-analysis has been performed. A key limitation of this systematic review is that only mortality outcomes and ICU admission rates were reported in most of the studies. Furthermore, not all other important clinical outcomes such as vital signs, lab results, mechanical ventilation, and vasopressor use were reported in all the studies. Therefore, the generalizability of these findings is not possible as results might be different if all the studies have reported all outcomes. Another limitation is the heterogeneity in outcome measures across the included studies. While the primary outcomes of interest were mortality and ICU admission, the criteria for these outcomes varied. For instance, some studies reported 30-day mortality, while others reported in-hospital mortality or long-term survival.

Recommendation and future research directions

Future research should focus on larger, prospective studies with longer follow-up periods to validate the findings and refine the use of CURB-65 and qSOFA in clinical settings.

## Conclusions

This systematic review highlights the strengths of the CURB-65 and qSOFA scoring systems in predicting pneumonia outcomes, with CURB-65 showing superior sensitivity and AUC for mortality prediction and qSOFA demonstrating better performance in predicting ICU admissions. These findings have significant implications for clinical practice, suggesting that clinicians could optimize patient assessment and management by using both scoring systems, each addressing different clinical needs, CURB-65 for identifying high-risk patients requiring intensive management and qSOFA for recognizing those who may need critical care interventions. 
